# Brief communication: reasons for non-adherence of co-trimoxazole prophylaxis therapy among people living with HIV in a resource-limited setting, Northern Ethiopia

**DOI:** 10.1186/s12981-024-00635-2

**Published:** 2024-08-05

**Authors:** Gebrehiwot Teklay, Meryem Mohammedbrhan, Desilu Mahari Desta

**Affiliations:** 1 Department of Clinical Pharmacy, School of Pharmacy, College of Health Sciences, Mekelle University, P.O.Box: 1871, Mekelle, Tigray, Ethiopia; 2 Department of Pharmacy, Quiha General Hospital, Mekelle, Tigray, Ethiopia

**Keywords:** Cotrimoxazole, Nonadherence, Reasons, People living with HIV

## Abstract

This study aimed to assess the prevalence and reasons for nonadherence to cotrimoxazole prophylaxis therapy. A cross-sectional study was conducted among people living with HIV attending Ayder Comprehensive Specialized Hospital. Data were collected through interviews and reviews of medical records. Binary logistic regression was employed to analyze factors associated with CPT nonadherence. Approximately two-thirds (65.5%) of the participants were non-adherent to co-trimoxazole prophylaxis therapy. The main reasons for non-adherence were side effects, pill fatigue and forgetfulness. Strategies to improve adherence to co-trimoxazole prophylaxis therapy should focus on the combined patient, clinical and medication related issues of people living with HIV.

## Background

The benefits of cotrimoxazole prophylaxis therapy (CPT) in reducing HIV related mortalities and morbidities associated with opportunistic infections are well demonstrated [1, 2]. Despite its effectiveness and ongoing implementation of the intervention for years, studies to explore the level of uptake and barriers to cotrimoxazole prophylaxis adherence over a course of antiretroviral therapy (ART) are necessary. Understanding the dynamics of adherence as people living with HIV (PLHIV) grow from adolescence into adulthood and eventually into old age will help optimize the management of HIV infection. The aim of this study was to assess the prevalence of CPT nonadherence and reasons for nonadherence among PLHIV in a resource-limited setting.

## Methods

This study was conducted at the HIV clinic of Ayder Comprehensive Specialized Hospital (ACSH), a public teaching hospital providing general inpatient and outpatient services [3]. The HIV clinic in ACSH provides ART for 1,440 PLHIV. PLHIV in this resource limited setting receive ART and medications for opportunistic infections free of charge. Data was collected from March to May 2020.

A cross-sectional study was conducted among adolescents and adults PLHIV who had been prescribed CPT between February and May 2020. Data collection involved interviews and a review of medical records. A pre-tested semi-structured questionnaire, containing both closed and open-ended questions was used to collect participants’ socio-demographic, clinical characteristics, adherence and reasons for non-adherence. Data regarding clinical data and treatments (CPT, ART regimens and other concurrent medications) were abstracted from medical records using a structured data abstraction format. Based on the data obtained from the ART clinic, there were about 780 PLHIV (N) eligible for CPT (with CD4 count ≤ 350 cells per ml). Using the formula for estimation of a single proportion [n_o_ = (Z α/2)^2^ p(1-p)/d^2^] where Z = standard normal variable at 95% confidence level (1.96), p = the prevalence of CPT non-adherence (assumed to be 50%) and finally adjusted using a correction formula for the finite population [n= (n_o_N)/(1+(n_o_ -1))], a sample of 177 was studied.

In this study, adherence to antiretroviral (ARV) medications and adherence to CPT was measured using patient self-reports based on a 30-day recall. Adherence was calculated as the proportion of pills taken to the number prescribed within 30 days. Participants with a self-report score of ≥ 95% were considered adherent.

The collected data was initially checked for completeness and consistency before being entered into the Statistical Package for Social Sciences (SPSS) version 20.0. Binary logistic regression was employed to analyze factors associated with CPT nonadherence. The results of the logistic regression were expressed as crude odds ratios (COR) and adjusted odds ratios (AOR) with 95% confidence intervals (CI). A *p* value of less than 0.05 was considered statistically significant.

## Results

In this study, 177 PLHIV participated, with nearly half (53.1%) of them being female and the majority (72.9%) were adults between the age of 31 and 59 years. Approximately half (53.7%) reported having family support both financially and in following their treatments. The Tenofovir (TDF)-based first-line regimen (TDF/3TC/DTG) was the most common type of ART regimen prescribed to about 63% of the participants. Regarding knowledge and attitudes about CPT, almost half (48.6%) of the respondents knew the benefits of CPT. However, only about 29% of the study participants believed that CPT was as important as ART.

We found that the prevalence of nonadherence to ART was 37.9%, while nonadherence to CPT was 65.5%. More than half (61.0%) of the participants experienced side effects during CPT, with gastrointestinal side effects, rash, fatigue and central nervous system side effects being reported by about 40%, 18%, 7% and 4.5% of participants, respectively. Participants who were nonadherent to CPT cited reasons such as side effects and/or pill fatigue (39.4%), forgetfulness (31.7%), preferring to take holy water (7.7%), feeling healthy (7.7%), fear of taking in front of others (7.7%), and not having food to take with (5.8%).

Univariate logistic regression revealed that variables such as CD4 count, adherence to ART, side effects, knowledge about the benefit of CPT and attitude regarding the importance of CPT were significantly associated with CPT nonadherence (*p* value < 0.05) (Table 1). However, in the multivariable binary logistic regression, only CD4 count, adherence to ART and side effects were significant predictors of nonadherence to CPT (Table 2). Participants with CD4 counts above 350 were 3.48 times more likely to be nonadherent to CPT than those with CD4 counts of 350 or less (*p* value = 0.002). Those who were adherent to ART were less likely to be nonadherent to CPT compared to nonadherent individuals (*p* value = 0.001). Additionally, participants who experienced side effects were 3.85 times more likely to be nonadherent to CPT (*p* value = 0.001). Though a significant number of participants believed CPT is less important than ART, the multivariable binary logistic regression did not show a statistically significant association with nonadherence to CPT.

**Table 1:** Univariate binary logistic regression of factors associated with CPT nonadherence among PLHIV in ACSH, June 2020 (*N* = 177)

Variables		CPT Nonadherent	CPT Adherent	COR (95% CI)	*p* value
Age	≤ 30	18/116 (15.5)	17/61 (27.9)	0.475 (0.224–1.009)	0.053
Sex	Male	51/116 (44.0)	32/61 (52.5)	1.406 (0.755–2.620)	0.283
Residence	Urban	100/116 (86.2)	53/61 (86.9)	1.060 (0.426–2.638)	0.900
Religion	Christian	111/116 (95.7)	56/61 (91.8)	0.505 (0.140–1.816)	0.295
Occupation	No job	35/116 (30.2)	16/61 (26.2)	0.823 (0.411–1.648)	0.582
Educational Level	No formal education	40/116 (34.5)	18/61 (29.5)	0.675 (0.270–1.691)	0.401
	Elementary/High school	58/116 (50.0)	31/61 (50.8)	0.802 (0.342–1.877)	0.611
Marital Status	Single/divorced/widowed	67/116 (57.8)	32/61 (52.5)	0.807 (0.433–1.505)	0.500
Family support	Yes	61/116 (52.6)	34/61 (55.7)	1.135 (0.609–2.117)	0.690
CD 4 counts	> 350	19/116 (16.4)	27/61 (44.3)	4.054 (2.003–8.205)	0.000*
Duration on ART treatment (years)	< 5	65/116 (56.0)	33/61 (54.1)	1.081 (0.580–2.016)	0.806
Co morbidities	Yes	21/116 (18.1)	6/61 (9.8)	0.494 (0.188–1.297)	0.152
Concurrent Medication	Yes	21/116 (18.1)	7/61 (11.5)	0.586 (0.234–1.469)	0.255
Type of ART regimen	TDF based	95/116 (81.9)	48/61(78.7)	0.589 (0.188–1.851)	0.365
	AZT based	14/116 (12.1)	7/61 (11.5)	0.583 (0.141–2.410)	0.456
Adherence to ART	Yes	86/116 (74.1)	24/61 (39.3)	0.226 (0.117–0.438)	0.000*
Side effect	Yes	32/116 (27.6)	37/61 (60.7)	4.047 (2.101–7.795)	0.000*
Knows the benefit of CPT	Yes	52/116 (44.8)	39/61 (64.0)	2.182 (1.153–4.129)	0.017*
Attitude towards CPT: CPT is just as important as ART	Agree	26/116 (22.4)	25/61 (41.0)	3.188 (1.504–6.759)	0.002*
	Not sure	27/116 (23.3)	17/61 (27.9)	2.088 (0.943–4.621)	0.069

* Indicating statistically significant association (*P* value < 0.05)

ART, Antiretroviral therapy; AZT, Zidovudine based (AZT-3TC-EFV); CPT, Cotrimoxazole prophylaxis therapy; COR, Crude Odds Ratio; CI, Confidence Interval; N, Number; TDF, Tenofovir based (TDF/3TC/DTG)

**Table 2:** Multivariable binary logistic regression of factors associated with CPT nonadherence among PLHIV in ACSH, June 2020 (*N* = 177)

Variables		CPT Nonadherent	CPT Adherent	AOR (95% CI)	*p* - value	
CD 4						
	> 350	19/116 (16.4)	27/61(44.3)	3.481 (1.555–7.792)	0.002**	
Adherence to ART						
	Yes	86/116 (74.1)	24/61 (39.3)	0.279 (0.128–0.608)	0.001**	
Side effects						
	Yes	32/116 (27.6)	37/61 (60.7)	3.849 (1.789–8.284)	0.001**	
Knows the benefit of CPT						
	Yes	52/116 (44.8)	39/61 (63.9)	0.483 (0.051–4.524)	0.523	
Attitude towards CPT: CPT is just as important as ART						
	Agree	26/116 (22.4)	25/61 (41.0)	4.831 (0.442–52.788)	0.197	
	Not sure	27/116 (23.3)	17/61 (27.9)	6.681 (0.720-61.961)	0.095	

** Indicating statistically significant association (*P* value < 0.05)

ART, Antiretroviral therapy; CPT, AOR, Adjusted Odds Ratio; Cotrimoxazole prophylaxis therapy; CD4, Clustered differentiation; CI, Confidence Interval; N, Number;

## Discussion

In this study, approximately two-thirds (65.5%) of the participants were non adherent to CPT. Although CPT is considered a feasible, cost-effective and well-tolerated intervention [4], side effects were a predictor and one of the main reasons for nonadherence to CPT in our study. The impact of medication side effects on adherence to long-term treatments is well documented by other studies [5, 6]. PLHIV adherent to ART were 72.1% less likely to be nonadherent to CPT compared to those nonadherent to ART (*p* value = 0.001), indicating a direct relationship between adherence to ART and CPT. However, it is unclear whether CPT nonadherence led to nonadherence to ART or vice versa. We found PLHIV with CD4 counts above 350 were 3.48 times more likely to interrupt CPT on their own than those with CD4 counts of 350 or less (*p* value = 0.002). This is because PLHIV with good immune response are more likely to receive information on when to discontinue CPT from their health care providers or from other PLHIV receiving care in the ART clinic. Similarly, Geresu et al. [7] also reported that a CD4 count above 350 was the primary reason for discontinuing CPT.

Despite the majority of PLHIV in this study having knowledge about the benefits of CPT, a significant number of participants believed that CPT is less important than ART. Though not statistically proven, these negative beliefs might be a driving factor for the high level of nonadherence to CPT. The relationship between patients’ knowledge of their treatment and improvement of adherence has been demonstrated in several studies [5, 6, 8].

This study has some limitations. Being a cross-sectional study with a small sample size, it was unable to establish causal relationships between certain variables. Future research should focus on investigating the impact of CPT nonadherence on ART outcomes.

This study conducted in a resource-limited setting has identified gaps in the uptake of CPT and barriers to adherence. PLHIV appear to undervalue the importance of CPT compared to ART. A significant number of PLHIV were found to be nonadherent to CPT due to reasons such as side effects, pill fatigue and forgetfulness, highlighting the need for ongoing medication counseling for PLHIV to emphasize the benefits of CPT.

## Data Availability

The data supporting the conclusions of this study are presented in the main manuscript. Any additional data can be obtained by contacting the corresponding author.

## Abbreviations

ACSH: Ayder Comprehensive Specialized Hospital
ART: Antiretroviral therapy
AOR: Adjusted Odds Ratio
AZT: Zidovudine
CD4: Clustered Differentiation
CI: Confidence Interval
CPT: Cotrimoxazole prophylaxis therapy
COR: Crude Odds Ratio
DTG: Dolutegravir
HIV: Human immunodeficiency Virus
N: Number
OIs: Opportunistic infections
PLHIV: People living with HIV
TDF: Tenofovir,3-TC, Lamivudine
